# Stephen B. Thacker, MD, MSc — 1947–2013

**Published:** 2013-03-01

Stephen B. Thacker, MD, MSc, a retired Assistant Surgeon General in the U.S. Public Health Service, and recent Director of the Office of Surveillance, Epidemiology, and Laboratory Services at CDC, died on February 15, 2013, in Atlanta, Georgia. He was 65. Dr. Thacker served with distinction in key CDC positions, including Deputy Director (Acting), during a career that spanned more than 36 years. He was the recipient of two of the U.S. Public Health Service’s highest individual honors, the Distinguished Service Medal and the Surgeon General’s Medal. Throughout his public service, he provided essential support to the *Morbidity and Mortality Weekly Report* (*MMWR*) series of publications.

Born in Independence, Missouri, Dr. Thacker was educated at Princeton University and the Mt. Sinai School of Medicine. He completed a residency in Family Medicine at Duke University and obtained an MSc degree at the London School of Hygiene and Tropical Medicine. He was board-certified in Family Medicine and Public Health and General Preventive Medicine. During his career, he published over 240 articles and book chapters on public health and health care.

Dr. Thacker joined CDC’s Epidemic Intelligence Service (EIS) Program in 1976 and was assigned to the Washington, DC, health department. Beginning with an early leadership role as director of CDC’s public health surveillance office and continuing throughout his career, he made substantive contributions to the public health practice of surveillance that have been institutionalized in the United States and globally. One example is the enduring effect of his influence on the *MMWR* series: in 1983, Dr. Thacker conceived and helped to launch the Surveillance Summary component of the *MMWR* series to advance the science and effectiveness of this essential function in U.S. public health practice.

**Figure:**
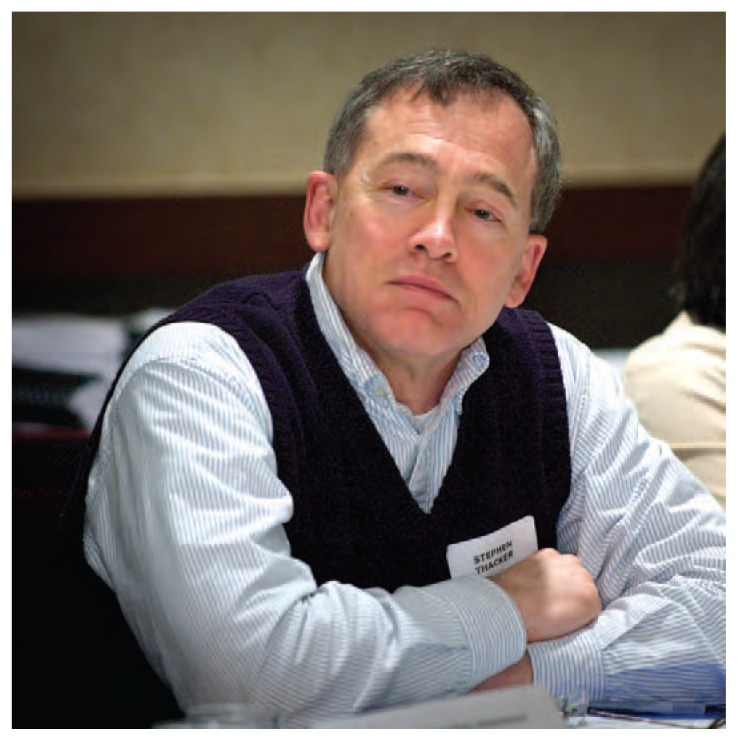
Dr. Thacker in 2005, leading the Symposium on Diversity, Leadership Development, and Succession Planning.

In addition to his contributions to *MMWR* through surveillance, Dr. Thacker provided ongoing, routine, senior-level review of the contents of each issue of the *MMWR* weekly throughout most of his career. His expertise in epidemiology, public health practice, and related disciplines, and his knowledge of and insights about CDC history were highly valued by *MMWR* editors and added substantially to the quality, accuracy, and integrity of the publication.

